# Characteristics of Activation Rate and Damage of Ion-Implanted Phosphorous in 4H-SiC after Different Annealing by Optical Absorption

**DOI:** 10.3390/mi13050804

**Published:** 2022-05-21

**Authors:** Jingmin Wu, Xiang Yang, Fengxuan Wang, Zhiyu Guo, Zhongchao Fan, Zhi He, Fuhua Yang

**Affiliations:** 1 Engineering Research Center for Semiconductor Integrated Technology, Institute of Semiconductors, Chinese Academy of Sciences, Beijing 100083, China; wujingmin@semi.ac.cn (J.W.); xyang@semi.ac.cn (X.Y.); wangfengxuan@semi.ac.cn (F.W.); gzy@semi.ac.cn (Z.G.); 2 College of Materials Science and Opto-Electronic Technology, University of Chinese Academy of Sciences, Beijing 100049, China; fhyang@semi.ac.cn; 3 School of Integrated Circuits, University of Chinese Academy of Sciences, Beijing 100049, China; 4 Institute of Microelectronics, Chinese Academy of Sciences, Beijing 100029, China; 5 State Key Laboratory for Superlattices and Microstructures, Institute of Semiconductors, Chinese Academy of Sciences, Beijing 100083, China

**Keywords:** laser annealing, thermal annealing, P^+^ ion implantation, ellipsometer, optical absorption

## Abstract

We investigated the ellipsometer-based characterization method being used to quickly evaluate the depth of the damage layer in ion-implanted 4H-SiC. This method had the advantages of low cost, convenience, and non-destructiveness. Optical absorption of n-type 4H-SiC substrate, P^+^ ion-implanted, laser-annealed, and conventional high-temperature annealed wafers were investigated at room temperature. Three peaks were observed in the absorption spectra collected for various samples. The degree of electrical activation after laser annealing or high-temperature annealing was evaluated qualitatively from the absorption peak intensity at 2.67 eV. The circular transmission line method (CTLM) results were consistent with the optical absorption results. However, it was found that the effective carrier concentration after laser annealing was significantly lower than that after high-temperature annealing.

## 1. Introduction

Silicon carbide (SiC) is drawing great attention in industry for its favorable properties in high-temperature and high-power applications. 4H-SiC is considered to be an ideal candidate material for power device applications, due to its excellent properties such as high critical electric field strength and high electron mobility [1,2]. Due to the superior physical properties of 4H-SiC, it has been also proposed for temperature sensors [3]. Power devices, such as Schottky-barrier diodes (SBD), junction field-effect transistor (JFET), and metal-oxide-semiconductor field-effect transistor (MOSFET) have been manufactured and commercialized in the market. The implantation/annealing process to create a pn junction is one of the most important processes in SiC device manufacturing. A good surface morphology, a low lattice defect density, and a high electrical activation rate in the implanted region are required to achieve excellent device performance. Usually, this can be realized by either conventional high-temperature annealing above 1500 °C or laser annealing after the implantation to activate the implanted species [4,5]. Furthermore, a C-cap or AlN cap is covered on the implanted sample, to protect the material surface during the annealing process [6].

Many methodologies can be used to characterize the crystalline materials after ion implantation. The ion implantation depth profile is usually measured by the secondary ion mass spectrometry (SIMS) measurements. The electrical properties of the implanted region are usually evaluated by the Hall and capacitive-voltage (CV) measurements. It is worth mentioning that the Hall measurement requires electrical isolation between the implanted region and substrate, as well as the patterned electrodes which need to be processed by lithographic and etching processes. CV measurement is not effective for an implanted sample with high doping concentration (>(1–2) × 10^19^ cm^−3^) due to the difficulty of forming a good Schottky contact [7]. Among various characterization methods, the ellipsometer is very sensitive to changes in the optical properties of thin films. As ion implantation significantly changes the optical properties of crystalline material, the damaged layer depth can be monitored non-destructively using an ellipsometer. Furthermore, the ability of semiconductors to absorb light of different wavelengths is another important optical property. The light absorption of SiC materials depends on the polytype, dopant, and doping concentration [8,9,10]. Based on these features, the activation of the implanted SiC crystal can be evaluated by the degree of the crystal damage recovery of SiC after laser annealing or high-temperature annealing, by comparing optical absorption characteristics.

## 2. Experiments

In this study, the N-doped (~10^18^ cm^−3^) (0001)-oriented commercial 4H-SiC substrate from Sicc Co. was implanted with phosphorous (P) ions. At higher doping concentrations (>(2–5) × 10^19^ cm^−^^3^), P is the preferred donor impurity in SiC because it has a higher electrical activation rate than nitrogen (N) [11]. To obtain a box implantation profile with a plateau of 3 × 10^20^ cm^−3^, the implantation process was set as follows: ion energy and ion doses - 380 keV/5.0 × 10^15^ cm^−2^, 250 keV/3.8 × 10^15^ cm^−2^, 140 keV/2.4 × 10^15^ cm^−2^, and 60 keV/1.6 × 10^15^ cm^−2^, respectively. Before post-implantation laser annealing, the implanted surface of each sample was covered with a carbon film to avoid Si evaporation during the annealing process. The C film was created by depositing the photoresist on a SiC wafer, which was then heated to 700 °C in the Ar atmosphere. During this process, the photoresist was pyrolyzed and transformed into an amorphous carbon material. An Nd:YLF pulsed laser (wavelength: 527 nm, pulse duration: 200 ns, repetition: 500 Hz) was used to irradiate the C-cap/4H-SiC. Laser radiation was conducted in the N_2_ atmosphere for further surface protection. The thermally activated sample with a carbon film was prepared by annealing at 1700 °C for 30 min. The carbon caps of all samples were subsequently removed by O_2_ plasma ashing. Then, the sputtered nickel metal layer was patterned by photolithography to form circular transfer length method (CTLM) test patterns. Finally, the samples were annealed at 1050 °C for 60 s in the N_2_ atmosphere to form a good ohmic contact. The samples were measured with an M-2000DI spectroscopic ellipsometer using angles of incidence of 60° and 70°. The optical absorption measurements were carried out in the wavelength range of 280–700 nm using Agilent Carry 7000 UV-Vis-NIR at room temperature. The specific contact resistivity of P^+^-implanted and annealed samples was calculated from CTLM test patterns [12]. Four types of samples were selected, which were: 4H-SiC substrate wafer (S); implanted wafer (I); laser annealed wafers (Ls); and thermally annealed wafer (H). The sample Ls were laser-annealed with energy from 1.7 to 2.2 J/cm^2^. 

## 3. Results and Discussion

**Figure 1:**
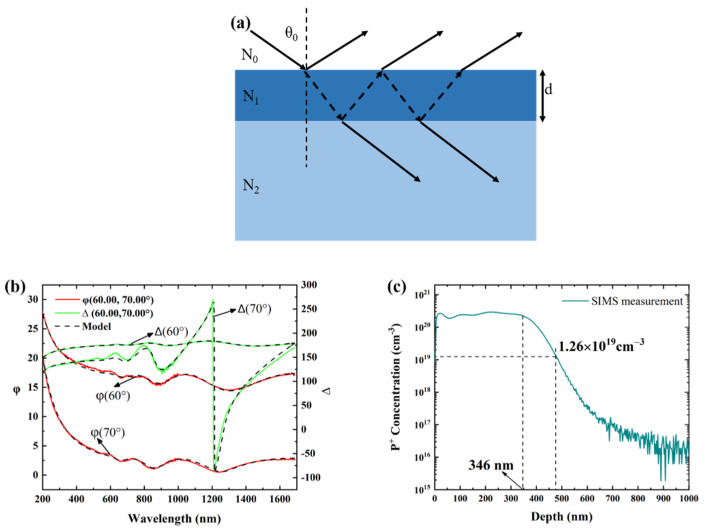
(**a**) Three-phase (ambient/film (i.e., ion−damaged layer)/substrate) optical model, where d is the film thickness and N is the refractive index; (**b**) Experimental (solid line) and fitted (dashed line) curves of the ion-implanted sample tested by ellipsometer, where φ and Δ are used as ellipsometry parameters; (**c**) Box profile of P ions implantation formed by multiple implants. The implantation profile was measured by SIMS.

Ellipsometry has proven to be a powerful technique for determining film thickness, refractive index (*n*), and extinction coefficient (*k*), by measuring polarization change (φ and Δ) when light reflects from the interface, where φ and Δ refer to the amplitude ratio and phase difference between the p-component and the s-component, respectively. To improve accuracy, the φ and Δ data were measured at multiple incident angles in the spectral range of 200–1700 nm. Since the ion implantation generated a damaged layer, the optical model consisted of a damaged layer, described by the Tauc–Lorentz oscillator [13], and a 4H-SiC substrate at the bottom, as shown in Figure 1a. Determination of ion-damaged thickness was achieved by modeling the ion-damaged layer with the oscillator. According to Fresnel’s law and Snell’s law, in a three-phase (see Figure 1a) optical model, the total reflection coefficient of the p- or s-component is described by [14]:

(1) $$r_{p\left(total\right)}=\frac{r_{p01}+r_{p12}e^{-i2\beta }}{1+r_{p01}r_{p12}e^{-i2\beta }}$$

(2) $$r_{s\left(total\right)}=\frac{r_{s01}+r_{s12}e^{-i2\beta }}{1+r_{s01}r_{s12}e^{-i2\beta }}$$

(3) $$d=\frac{\beta \lambda }{2\pi \sqrt{N_{1}^{2}-N_{0}^{2}{\left(\sin\theta_{0}\right)}^{2}}}$$

where *β* is the phase difference between two adjacent reflected beams, *r_p_*_01_ and *r_s_*_01_ are the reflection coefficient of the p- or s-component, respectively, at the air/film interface, *r_p_*_12_ and *r_s_*_12_ are the reflection coefficient of the p- or s-component, respectively, at the film/substrate interface, *d* is the film thickness, *θ*_0_ is the incident angle, and *N*_0_ and *N*_1_ are the refractive indexes of air and film, respectively.

The measured φ and Δ are described by the ellipsometry fundamental equation:

(4) $$\frac{r_{p}}{r_{s}}=\tan\varphi e^{j\Delta }$$

Taking into account the non-uniformity of the optical parameters of the damaged layer, caused by ion implantation along the film thickness direction, the optical model was modified by adding a grade [15]. Grading is used to divide the ion-damaged layer into a series of sublayers with different optical constants. The φ and Δ can be simulated by CompleteEASE software using the optical model and Equations (1)–(4). The measured φ and Δ of the P^+^-implanted sample were well-fitted, as shown in Figure 1b. The thickness of the ion-damaged 4H-SiC layer in sample I was determined to be 476 nm. After P^+^ ion implantation, the plateau width of the box-shaped concentration distribution measured by SIMS was 346 nm (see Figure 1c). Then the concentration gradually decreased, forming a concentration tailing region. 476 nm determined by the ellipsometer is where the P^+^ ions concentration of 1.26 × 10^19^ cm^−3^ —which was higher than the doping concentration of the 4H-SiC substrate—was located (see Figure 1c). SiC lattice damage caused by high concentration of P^+^ ions can still change the optical properties of the material, which can be detected by an ellipsometer. Therefore, an ellipsometer can be used non-destructively and rapidly to detect and characterize the thickness of the damage layer caused by ion implantation.

**Figure 2:**
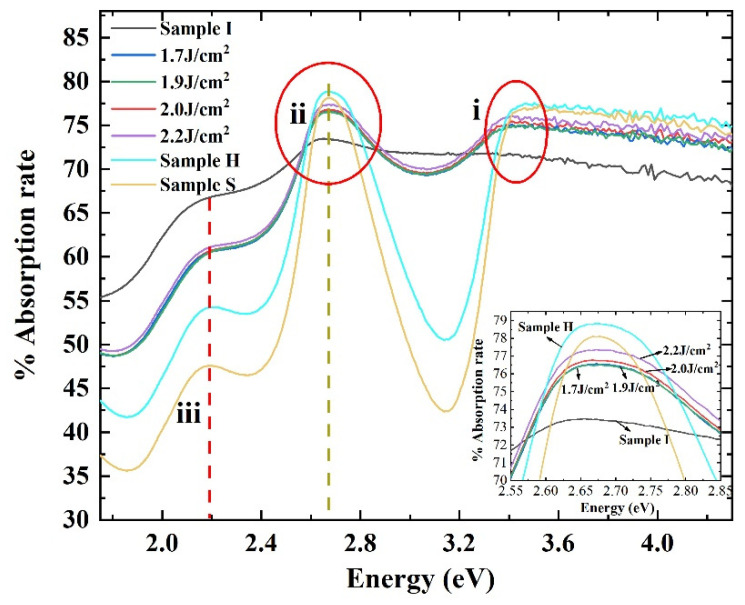
Absorption spectra of four selected 4H-SiC samples measured at room temperature. They are n-type 4H-SiC substrate (S-solid yellow line), P^+^ ions implanted sample (I-solid black line), laser-annealed samples at different energy densities (Ls), and high-temperature annealed sample (H-solid cyan line): (i) represents absorption from valence band to conduction; (ii) from donor level or lowest conduction band to higher conduction band; (iii) from the defect states. The inset is an enlarged view of the absorption peak (ii).

It is well known that the optical absorption of SiC materials depends on SiC polymorphism [9] and dopants [16]. The absorption intensity (A) of crystalline material can be determined from the measured transmittance (T) and reflectance (R), i.e., A(λ) = 100% − T(λ) − R(λ). The influence of surface scattering on reflectance can be investigated using a formula devised by Engelbrecht, et al. [17]:

(5) $$R=R_{0}\exp\left\{\left(-16\pi^{2}\delta^{2}\right)/\gamma^{2}\right\}$$

where γ and δ are root mean square and incident wavelength, respectively, *R*_0_ is original reflectance, and *R* is corrected reflectance. The surface roughness of samples S, I, Ls, and H, measured by AFM, was less than 0.5 nm. The corrected *R* was equal to *R*_0_ according to Equation (5) under the influence of this roughness. Hence, surface roughness had little effect on the test results. All absorption spectra with features (i), (ii), and (iii) are shown in Figure 2. 

In Figure 2, the characteristic peak (i) corresponds to the transition of electrons from the valence band to the conduction band. Ion implantation generates a large number of point defects, which all create localized levels within the bandgap, such as deep levels [18,19], resulting in a decrease in the intensity of the peak (i). The damage center introduces local non-uniform strain, and provides a scattering center for electrons, which tends to broaden the optical structure [20]. As one can see from the diagram, the characteristic peak (i) after P^+^ ions implantation became wider, and the intensity declined, compared with the sample S. Feature (i) appeared after PLA or high-temperature annealing, indicating that the damage caused by ion implantation is gradually recovered after different annealing. However, the absorption intensity of sample Ls was lower than that of samples S and H. This demonstrated that there were still significant residual defects in the sample Ls after PLA. 

The absorption peak (ii) is present at 2.67 eV in sample S. This absorption band has been discussed by many researchers. Wellmann, et al. [10], Limpijumonong, et al. [8], and Weingärtner, et al. [21] believe that the absorption band (ii) is related to the transition of electrons from the donor level or conduction band to a higher conduction band. A large number of point defects caused by the ion implantation reduced the effective carrier concentration. Eventually, the absorption intensity of the peak (ii) in Sample I declined. After the PLA process, the intensity of peak (ii) gradually increased with increasing laser energy density (see inset of Figure 2). This indicates that the higher the laser energy density is, the higher the effective carrier concentration will be. The result for sample Ls in Figure 2 shows that the intensity of peak (ii) after laser annealing at 2.2 J/cm^2^ was higher than that of sample I, but lower than that of the 4H-SiC substrate and that of the standard high-temperature annealed sample. Thus, it could be concluded that the electrical activation efficiency of the laser-annealed sample was lower than that of the high-temperature annealed sample; for sample H, its intensity was higher than that of sample S, suggesting that the carrier concentration after high temperature annealing is higher than the 4H-SiC substrate. 

The last characteristic peak (iii) marked could be attributed to the defect states in the bandgap [22]. It can be seen that the intensity of sample I at peak (iii) increased significantly after ion implantation, compared with the substrate (sample S). After different annealing treatments, the intensities of samples Ls and H at peak iii started to decrease, while the intensity of the laser-annealed sample was higher than that of the high-temperature annealed sample. These results reveal that the recovery of lattice damage by laser annealing is inferior to that by standard high-temperature annealing. It is worth noting that the intensity of peak (iii) for sample H was higher than that for sample S. This may have been due to the generation of defects during high-temperature annealing, resulting in extended defects such as dislocation loops [23] or defect levels with thermal stability [19]. 

**Figure 3:**
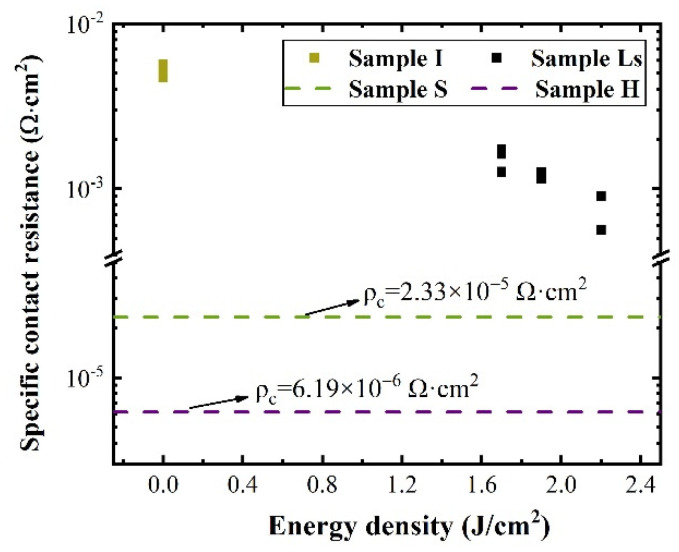
Specific contact resistivity as a function of energy density.

The CTLM was used to calculate the specific contact resistivity ($\rho_{c}$) of the four types of samples. The $\rho_{c}$ of the ohmic contact achieved by the tunneling current was proportional to $\exp\left\{\left(\emptyset_{B}\right)/N_{d}^{1/2}\right\}$. Therefore, the degree of electrical activation of the samples was indirectly verifiable by the $\rho_{c}$. Figure 3 shows the laser energy density dependence of the $\rho_{c}$ for the laser-annealed sample in this study. This figure shows that the $\rho_{c}$ values of sample Ls decreased sharply compared with that of sample I, and further decreased with the increase of the laser energy density. The P^+^-implanted impurities in 4H-SiC after PLA were electrically activated, and the activation rate increased with the increase of laser fluence. However, the contact resistance of the sample Ls after PLA at 2.2 J/cm^2^ was still higher than that of the n-type 4H-SiC substrate (2.33 × 10^−5^ Ω∙cm^2^). The $\rho_{c}$ of the sample H after high-temperature annealing reached as low as 6.19 × 10^−6^ Ω∙cm^2^ (see Figure 3). This electrical result was consistent with that of the absorption band (ii).

## 4. Conclusions

In conclusion, the depth of the ion implantation damaged layer was determined quickly and non-destructively by an ellipsometer. The obtained thickness was in compliance with the SIMS result. In addition, the optical absorption effect (bands (i)–(iii)) was used to study laser-annealed and thermally-annealed 4H-SiC samples after the implantation. In this study, the absorption peak (ii) intensity at 2.67 eV, which was related to the effective doping concentration, increased with the laser fluence. However, the intensity of the absorption peak (ii) after laser annealing at 1.7–2.2 J/cm^2^ was lower than that after standard high-temperature annealing. This phenomenon revealed that the electrical activation rate after laser annealing was lower than that after high-temperature annealing. Meanwhile, the high absorption peak after laser annealing at 2.19 eV demonstrated that there were still a lot of residual defects in the sample Ls. The $\rho_{c}$ value measured by the CTLM was about 1.7 × 10^−3^–5.6 × 10^−4^ Ω·cm^2^ after laser annealing at different energy densities. The minimum value was 6.19 × 10^−6^ Ω∙cm^2^ after high-temperature annealing. The CTLM results basically supported the optical absorption results.

The present work provides a convenient technique for monitoring the depth and uniformity of ion-implanted damaged layers, the degree of damage caused by ion implantation, and the degree of electrical activation after annealing.

## Data Availability

Not applicable.
